# Does it matter what is trained? A randomized controlled trial evaluating the specificity of alpha/delta ratio neurofeedback in reducing tinnitus symptoms

**DOI:** 10.1093/braincomms/fcad185

**Published:** 2023-06-26

**Authors:** Martin Jensen, Jose Carlos Garcia Alanis, Eva Hüttenrauch, Matilde Winther-Jensen, Mira-Lynn Chavanon, Gerhard Andersson, Cornelia Weise

**Affiliations:** Division of Clinical Psychology and Psychotherapy, Department of Psychology, Philipps University Marburg, 35032 Marburg, Germany; Eriksholm Research Centre, 3070 Snekkersten, Denmark; Copenhagen Hearing and Balance Center, Department of Otorhinolaryngology and Audiology, Rigshospitalet, 2100 Copenhagen, Denmark; Division of Clinical Child and Adolescent Psychology, Department of Psychology, Philipps University Marburg, 35032 Marburg, Germany; Division of Clinical Psychology and Psychotherapy, Department of Psychology, Philipps University Marburg, 35032 Marburg, Germany; Department of Data, Biostatistics and Pharmacoepidemiology, Centre for Clinical Research and Prevention, Copenhagen University Hospital Bispebjerg-Frederiksberg, 2000 Copenhagen, Denmark; Division of Clinical Child and Adolescent Psychology, Department of Psychology, Philipps University Marburg, 35032 Marburg, Germany; Department of Behavioural Sciences and Learning, Linköping University, 58183 Linkoping, Sweden; Division of Clinical Psychology and Psychotherapy, Department of Psychology, Philipps University Marburg, 35032 Marburg, Germany

**Keywords:** tinnitus, neurofeedback, EEG, alpha, delta

## Abstract

Previous studies showed that alpha/delta ratio neurofeedback was effective in reducing unpleasant psychological, emotional and perceptual consequences of tinnitus. The main goal of the present study was to investigate, whether the specific combination of enhancing alpha frequency band activity and reducing delta frequency band activity was necessary, or merely sufficient, to obtain a positive treatment outcome regarding tinnitus distress and intensity. A second research aim was to assess the relative contribution of neurofeedback-related non-specific and general non-specific effects in neurofeedback treatment. In a three-arm, randomized controlled trial, 94 chronic tinnitus patients were randomly assigned to one of three conditions: alpha/delta ratio neurofeedback (*n* = 31), beta/theta ratio neurofeedback (*n* = 28) and non-neurofeedback minimal treatment intervention (*n* = 35). Neurofeedback participants underwent 10 treatment sessions over a 4-week period. Outcome measures were collected pre-, mid- and post-interventions and at 3-months follow-up. The Tinnitus Handicap Inventory and the Tinnitus Magnitude Index were used as primary outcome measures for tinnitus distress and tinnitus intensity. EEG data recorded during training supplemented primary outcomes. Since data were repeated measures, the analyses used a two-level mixed effects model approach including by-subject random effects (random intercept). For the Tinnitus Handicap Inventory, the results showed no interaction effect. For the Tinnitus Magnitude Index, the analysis showed a significant time × group interaction, indicating that both alpha/delta ratio neurofeedback and beta/theta ratio neurofeedback reported reduced tinnitus intensity. Analysis of EEG data showed a consistent pattern for the alpha/delta ratio over the course of training. Compared to beta/theta ratio neurofeedback, alpha/delta ratio neurofeedback showed an elevated response. Conversely, for the beta ratio to theta ratio, the pattern was more inconsistent, with no clear indication of superiority for beta/theta ratio neurofeedback over alpha/delta ratio neurofeedback. The main question of this piece of research was whether alpha/delta ratio neurofeedback demonstrated frequency band specificity in the alleviation of tinnitus distress and perceived intensity. Results showed that alpha/delta ratio neurofeedback was sufficient but importantly ‘not’ necessary to achieve a positive outcome on both the Tinnitus Handicap Inventory and Tinnitus Magnitude Index, when compared to beta/theta ratio neurofeedback. Still, the data suggest a trend towards specificity for alpha/delta ratio neurofeedback. Because of this, it may be too premature to discard alpha/delta ratio neurofeedback in the treatment of tinnitus. Recommendations for future studies are outlined.

## Teaser

Jensen *et al*. report that, although there was a trend in the expected direction, alpha/delta ratio neurofeedback (ADR-NF) training did not show evidence of specificity. Still, the findings are encouraging enough to warrant future studies, which seek to advance our understanding of factors that contribute to ADR-NF treatment efficacy.

## Graphical Abstract

**Graphical Abstract:**
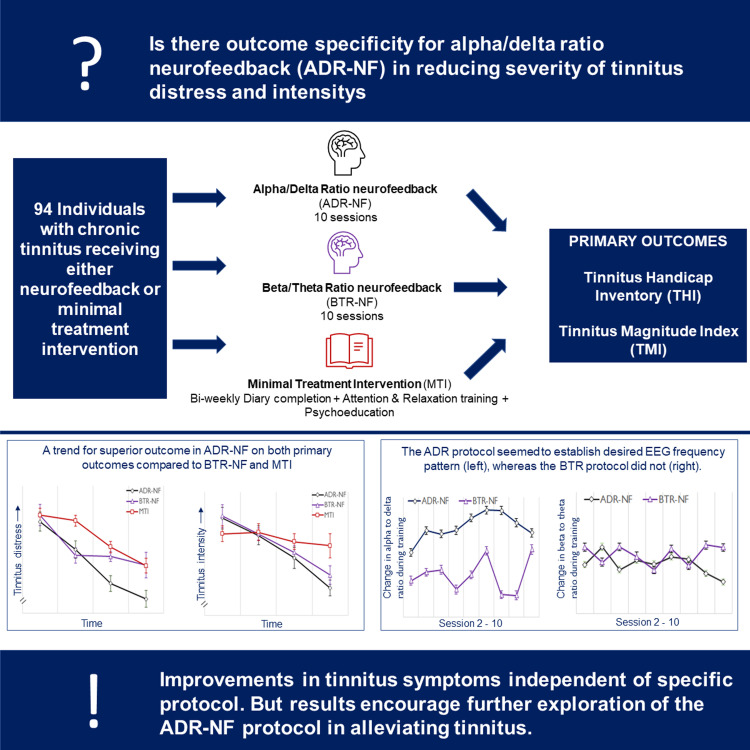

See Kleinjung, Meyer and Neff (https://doi.org/10.1093/braincomms/fcad209) for a scientific commentary on this article.

## Introduction

Defined by various sources as a ringing in the ears or a phantom sound with no external origin, tinnitus is a quite common phenomenon. It is experienced by large parts of populations across cultures and countries. When using the most common definition of having experienced tinnitus, i.e. as lasting more than 5 minutes at the time, studies suggest prevalence rates of between 11.9% and 30.3%.^1^ Fortunately, most individuals with tinnitus consider it a mild and only minor nuisance. Still, epidemiological surveys show that the condition causes severe distress for a significant number of tinnitus sufferers (e.g. Bhatt *et al*.^2^), and implies high societal costs.^3^ Thus, when additionally considering that tinnitus prevalence is on the rise,^4^ there is a need to explore new treatments, which might become increasingly important in the future.

Currently, the most effective management strategy in tinnitus is Cognitive Behavioural Therapy^5–8^ (CBT), in which people learn to cope with the negative impact of tinnitus on cognition and emotions. Yet, despite of CBT’s success in reducing tinnitus-related distress, the treatment does not achieve changes in tinnitus loudness; something that is much desired and hoped for by patients.^9^ Thus, to come closer to meeting these patient expectations, future treatments should not only aim for a reduction in tinnitus distress but also be directed at the source of the disturbance causing tinnitus: more specifically, aberrant brain activity.

There is considerable evidence that tinnitus arises from neurophysiological disturbances. Although not yet fully understood, these seem to involve several central auditory- and non-auditory structures, including the Dorsal Cochlear Nucleus and the limbic system (for a detailed review, see Henry *et al*.^10^, Rauschecker *et al*.^11^, Adjamian^12^ and Sedley *et al*.^13^). For this reason, it has been suggested that future treatments are likely those involving neuromodulation of neurophysiological activity.^14^

In the recent decade, EEG neurofeedback (EEG-NF) has emerged, as a treatment holding promise, in ameliorating both tinnitus distress and perceived loudness.^15–18^ Generally, EEG-NF is a biofeedback procedure, which is guided by the principle, that it is possible to gain volitional control over extracted features of EEG brain activity, by means of operant conditioning.^19,20^ Consequently, it is assumed that aberrant EEG activity associated with psychopathology can be normalized. This in turn leads to improvement in e.g. clinical symptomology (for review, see Niv^21^).

A model of tinnitus that has sparked some activity within neurofeedback research is the synchronization by loss of inhibition model (SLIM).^22^ Comparing resting-state brain activity in a tinnitus versus control group, the authors found enhanced delta and reduced alpha frequency EEG activity over temporal regions of the cortex. This discovery led to the development of a neurofeedback training protocol,^23^ which aims to increase the alpha (8–12 Hz) to delta (2–4 Hz) ratio (ADR). According to this specific protocol, training electrodes are placed frontocentrally at FC1, FC2, F3 and F4 of the 10/10 electrode system.^24^ In three exploratory trials, the ADR neurofeedback (ADR-NF) protocol was found effective in alleviating both tinnitus distress, and importantly, the self-reported loudness.^16,17,25^ Although encouraging, the main limitation in these studies was an absence of control groups. Consequently, little is known about the therapeutic mechanisms of the ADR-NF protocol, especially in relation to outcome specificity.

In the context of neurofeedback, outcome specificity is shown when ‘[…] the manipulation of a particular brainwave frequency at a particular electrode location is necessary for successful treatment outcome’.^26^ The main goal of the present study was therefore to assess outcome specificity for the ADR-NF protocol. To this end, it was compared to a protocol, in which participants sought to upregulate beta EEG activity and simultaneously downregulate theta EEG activity [beta/theta ratio neurofeedback (BTR-NF)]. This protocol was chosen, as at the time of study inception, no association between enhanced theta activity and reduced beta activity over temporal regions had been related to tinnitus. For this reason, it was considered an appropriate neurofeedback control condition (see also Sorger *et al.*^27^ and Enriquez-Geppert *et al.*^28^). In keeping all factors except neural targets identical between the two neurofeedback groups, the specificity of ADR-NF training could be assessed.

When adapting this approach to assessing outcome specificity, this trial depends on the assumptions of SLIM,^29^ i.e. that tinnitus is in fact associated with deviating alpha neurophysiological activity and delta neurophysiological activity over temporal cortices. This is not a universal finding.^29^ Nonetheless, since three studies have already published positive results with the ADR-NF protocol, it is considered both prudent and logical to evaluate it against a different protocol. This will give an indication of whether the ADR-NF protocol should be further explored and matured in subsequent trials.

**Figure 1:**
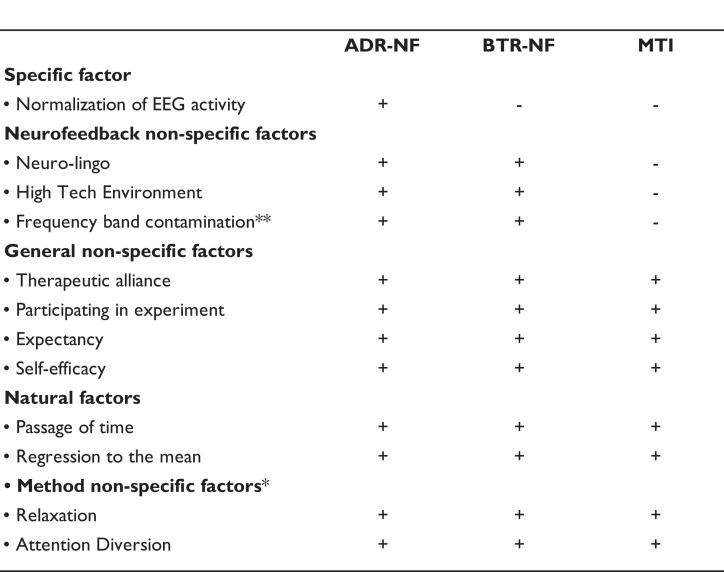
**Therapeutic factors in neurofeedback treatment**. Figure 1 shows an overview of factors suggested to contribute to clinical outcome in neurofeedback.^33^ ‘+’ indicates that the component contributes actively to clinical outcome in the present study. ‘−’ signifies that the factor has no impact on clinical outcome. *Not part of Ros *et al*.^33^ Adapted with permission from Ros *et al*.^33^

A secondary objective in this study was to investigate the extent to which neurofeedback non-specific- and general non-specific factors influence clinical outcome. There is a controversy in the literature with critics (e.g. Thibault *et al*.^30^, Schabus *et al*.^31^ and Schönenberg *et al*.^32^) advancing the idea, which most of the therapeutic benefits of neurofeedback are derived mainly from effects not related to self-regulating brain activity, but rather to neurofeedback non-specific factors or general non-specific factors (e.g. advanced neuro-lingo and a high technology environment; therapeutic alliance). A recent consensus paper^33^ called for studies that could disentangle these neurofeedback non-specific factors from both general non-specific factors (e.g. the therapeutic alliance and expectancy effects) and specific factors (self-regulation of neural circuits).^33^ With the introduction of a third non-neurofeedback intervention arm, specifically the minimal treatment intervention (MTI), we hoped to further our understanding of factors, which contribute to neurofeedback treatment effectiveness (for a comprehensive description of components in each of our three conditions, please see the study protocol. Note that the MTI is referred to as Diary Control Group in study protocol).^34^ To sum up, Fig. 1 illustrates which specific- and non-specific factors might contribute to clinical outcomes in the present study for each of the three intervention groups.

In putting it all together; based on the assumptions of SLIM,^22^ it was predicted (hypothesis 1) that ADR-NF would result in greater improvement, when it was compared to BTR-NF, on the two primary outcome measures used in the study, i.e. the Tinnitus Handicap Inventory (THI)^35^ and the Tinnitus Magnitude Index (TMI)^36^.

Since non-specific effects are considered especially potent in neurofeedback,^31^ it was furthermore predicted (hypothesis 2) that the two neurofeedback groups would exhibit superior clinical responses, when compared to the MTI at post-intervention and follow-up, with the greatest treatment response in ADR-NF.

## Materials and methods

A detailed description of the study process, the recruitment- and randomization procedure, the experimental design and the rationale behind the neurofeedback and MTI groups can all be found in the study protocol.^34^ The most important aspects of it are summarized in the following sections.

Data collection for the study took place at the Philipps-University Marburg in the period between May 2019 and June 2020. Ethics approval was obtained from both the Department of Psychology (ID 2018-4k) and the Department of Medicine at Philipps University (ID 162/18). The trial was registered with clinicaltrials.gov (ClinicalTrials.gov Identifier: NCT03550430).

### Participants

A total sample of 98 participants was estimated to adequately detect a medium assessment × treatment interaction.^34^ Because of the COVID-19 pandemic and the impact of restrictions on patient contact, we had to terminate data collection in March 2020. Consequently, our final sample consisted of 94 participants with chronic tinnitus. This number of participants made the study slightly underpowered in the intention-to-treat (ITT) analysis (ITT, *n* = 94, *β* = 0.78^37^). The ADR-NF group included 31 participants (*M*_age_ = 52.65, SD_age_ = 12.53, 11 females). A total of 28 participants were randomized to receive BTR-NF (*M*_age_ = 51.25, SD_age_ = 12.44, 10 females). The MTI group consisted of 35 participants (*M*_age_ = 49.71, SD_age_ = 15.41, 16 females). Since randomization was based on stratification^38^ (age, gender and tinnitus distress), the groups were equal on all key baseline characteristics. Table 1 shows baseline characteristics (upper panel) and frequency distribution (lower panel) for the sample. None of the comparisons showed differences between the groups (see Supplementary Table 1 for more audiological characteristics).

**Table 1:** Baseline sample characteristics for ITT and frequency distribution

		ADR-NF		BTR-NF		MTI			
Baseline characteristics	Total *n*	Mean	SD	Mean	SD	Mean	SD	*F*	*P*
Age	94	52.65	12.53	51.25	12.44	49.71	15.41	0.38	0.69
Tinnitus characteristics									
THI	94	36.58	17.63	38.25	17.20	37.20	17.65	0.42	0.96
TMI	94	54.73	20.50	55.19	21.40	51.56	20.88	0.29	0.75
Age onset	83	46.24	15.00	39.44	15.09	39.33	19.46	1.59	0.21
Duration (months)	90	146	114	163	150	126	94	0.73	0.49
Loudness match (dB)	93	51	22	52	23	42	22	2.28	0.11
PTA 4 kHz left^a^	93	15	14	15	14	13	12	0.24	0.79
PTA 4 kHz right^a^	93	12	11	14	13	12	10	0.21	0.80
Depression/sleep/treatment credibility									
Patient Health Questionnaire-9	94	6.32	3.77	6.50	3.78	7.20	3.95	0.50	0.61
Insomnia Severity Index	94	10.26	4.82	10.39	5.25	11.80	5.93	0.83	0.44
Credibility and Expectancy Questionnaire	90	32.85	7.52	31.51	8.15	29.85	6.15	1.35	0.27

Frequency distribution		*n*	%	*n*	%	*n*	%	χ^2^	*P*
Gender								0.94	0.62
Male	57	20	21	18	19	19	20		
Female	37	11	12	10	11	16	17		
Tinnitus pervasiveness								3.21	0.20
Constantly perceived	78	28	30	24	26	26	28		
Intermittent breaks in percept	16	3	3	4	4	9	10		

Top panel shows baseline characteristics of the intention-to-treat (ITT). Lower panel depicts frequency distribution.

ADR-NF, alpha/delta neurofeedback; BTR-NF, beta/theta ratio neurofeedback; MTI, minimal treatment intervention; dB, decibel; PTA, pure tone average.

a Average hearing threshold (500 hZ, 1 kHz, 2 kHz, 3 kHz, 4 kHz). For other audiological baseline characteristics, please see Supplementary Table 1.

### Procedure

In the pre-screening phase, participants were informed about the study, gave informed consent for questionnaire completion and were screened for principal eligibility. This was done via online screening questionnaires and structured telephone interviews. Both were based on the international statistical classification of diseases and related health problems (ICD-10)^39^. On their subsequent first visit to the laboratory, principally eligible participants signed an informed consent and took two attention tests (attention network test^40^ and sustained attention to response task^41^). On the same day, participants completed the pre-intervention (T_1_) questionnaires. Next, participants underwent an audiological assessment at the ENT department of the University Clinic Gießen and Marburg. When no objective cause for tinnitus was found, participants proceeded to the randomization phase, performed by an independent researcher. Based on this, participants were allocated to receive either ADR-NF, BTR-NF or MTI. Further details on the procedure can be found in the study protocol^34^ (see Supplementary Table 2 for inclusion and exclusion criteria).

### ADR-NF and BTR-NF

Over the 4-week intervention period, participants in both groups underwent 10 neurofeedback training sessions, with two to three sessions per week. As described in greater detail in the study protocol,^34^ EEG data was acquired with four frontocentral electrodes, positioned at FC1, FC2, F3 and F4 of the 10/10 electrode system.^24^ These electrode positions were chosen to maximize comparability with previously published ADR-NF studies.^16,17,42^

The first five sessions consisted of four training blocks. From session six onwards, a fifth block with transfer trials was introduced. Progressively, more individual trials in block five consisted of transfer trials. In session six, only 2 of 10 trials were transfer trials. Session seven had four transfer trials in block five. In session 10, all trials in block five were transfer trials (for detailed description, see Jensen *et al*.^34^). Participants were not provided with any instructions on mental strategies to use during neurofeedback training.

Mid-point assessments (T_2_) were completed after five training sessions and post-intervention (T_3_) questionnaires after 10 sessions. In the week following post-intervention (T_3_), participants returned to the department once more to take the attention tests (the results of which were published elsewhere^43^). Lastly, 12 weeks after T_3_, participants completed the follow-up questionnaires (T_4_). Afterwards, those who were interested received a neurofeedback booster session (not included in the analyses).

### MTI

**Figure 2:**
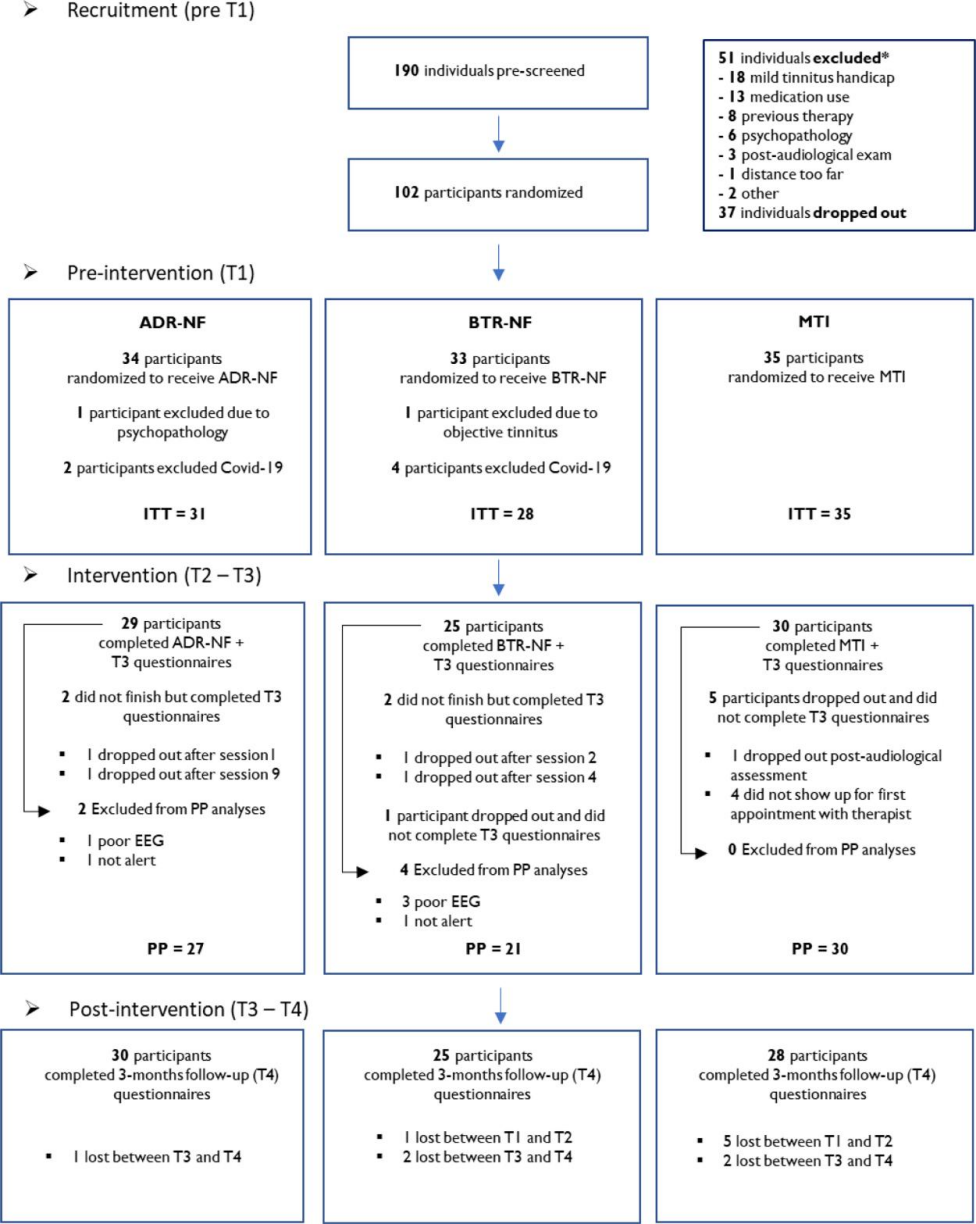
**Flowchart of the randomized controlled trial**. Figure 2 shows the flow of participants throughout the study period. *Please see study protocol^34^ for exclusion criteria (or Supplementary Table 2).

The MTI group had two face-to-face visits with a therapist within a 4-week period: an interval mirroring the intervention schedule in the neurofeedback groups. During the intervention period, participants were contacted by telephone twice by the therapist. The first time was 10 days after the first visit, and the second time was 17 days after Visit 1. Mid-point assessment (T_2_) was completed 2 weeks after the first therapist visit; post-intervention assessment (T_3_) was completed following the second therapist visit (4 weeks after first visit). Lastly, follow-up questionnaires (T_4_) were completed 16 weeks after the first visit. Figure 2 shows the flow of participants through the trial period, including reasons for drop-out/exclusion.

#### Primary and secondary outcome measures

Two primary outcome measures were predefined in the study protocol.^34^ Tinnitus distress was assessed with the THI^35^. The TMI^36^ was used to assess the subjectively rated tinnitus intensity. This instrument was included, as previous studies showed difficulties in receiving a reliable assessment of tinnitus loudness.^44^ For the present study, the TMI was standardized to a 0–100 score scale. Both primary outcome measures showed good internal consistency in the present study with THI (α = 0.95) and TMI (α = 0.88). For secondary and exploratory outcomes, the Tinnitus Functional Index^45,46^ (TFI) was chosen to assess changes in tinnitus functional impairment. For insomnia, the Insomnia Severity Index (ISI)^47^ was used, and for depression, the Patient Health Questionnaire-9 (PHQ-9).^48,49^ For exploratory purposes, analyses were performed on the ADR- and BTR-EEG data, as were participants perception of treatment credibility and expectations with the treatment Credibility and Expectancy Questionnaire (CEQ)^50^.

### Data analyses

#### Statistical model and main analyses

The main analyses were conducted on the ITT population, which included all randomized participants, who completed (T_1_) pre-intervention. Moreover, supplementary analyses were conducted on the per-protocol population (PP, *n* = 78, see Fig. 2 flowchart). All statistical analyses were carried out in the R programming environment.^51^

To investigate whether ADR-NF significantly differed from BTR-NF (hypothesis 1) and ADR-NF/BTR-NF from MTI (hypothesis 2), primary and secondary outcome data were analysed using a two-level mixed effects model approach including by-subject random effects (i.e. all models estimated a random intercept for each subject to account for repeated measures nested within participants). The data were modelled as a function of Group (fixed categorical with three levels between-subjects factor: ADR-NF, BTR-NF and MTI), Time (fixed categorical predictor with four levels within-subjects; T_1_ at 0 weeks, T_2_ at 2 weeks, T_3_ at 4 weeks and T_4_ at 16 weeks). In addition, as reported in the study protocol,^34^ the subject’s gender, age, reported treatment credibility and expectation (CEQ), interaction between group and time and the subjects’ THI baseline scores were introduced as covariates. This was done to account for individual variation (e.g. gender and initial tinnitus-related impairment) at the beginning of the study. Because the interaction term is included, the interpretation of coefficients changes. Consequently, the interpretation for the coefficient for the effect of ‘time’ relates to time in the baseline group, i.e. to MTI or BTR-NF. Coefficients for group alone can be interpreted as the effect of group allocation at baseline, which is not relevant in this study. When an interaction term is included, estimates for time and session alone cannot be interpreted as main effects; they must be interpreted as the effect for the group set as baseline in the interaction.

The R package ‘nlme’^52^ was used to model mixed effects. Both MTI and BTR-NF were used as reference in different analyses; MTI because it was considered the group closest to treatment as usual, and BTR-NF because of its comparison with ADR-NF. All categorical variables were deviation coded (i.e. effect coded), and all continuous variables were grand mean centred around zero prior to analysis. Since some individuals randomized at T_1_ did not continue in the study, missing variables were present in outcomes. Since missing outcome data were present, complete case analysis was rendered impossible. Therefore, multiple imputation was carried out on the ITT population, using the R package ‘mice’.^53^ Correlation structure of the mixed effects model was assessed in the complete case population by running models with different correlation structures on primary outcomes and choosing the model with lowest Akaike’s Information Criterion. A general correlation matrix was chosen as correlation structure. Prediction matrix was set up with a random intercept, and the imputation method chosen based on data type. The imputation model was set to produce 100 imputed datasets, and we report pooled estimates with standard errors from these 100 datasets. As secondary outcomes ISI and PHQ were not measured at T_2_, these were imputed in a separate model, while THI, TMI and TFI were imputed in the same model. The postulated two-way interaction between the variables Group and Time was further probed using two-tailed, planned contrasts in the per-protocol population (see study protocol for elaboration on the data analyses^34^).

#### Spectral analyses

As described in greater detail in the study protocol^34^ and in brief earlier, neurofeedback training blocks from session 6 to 10 included transfer trials in training block five. For the analysis, we included only training blocks with feedback, i.e. blocks one to four in each of the 10 sessions; an approach used previously.^31^

Offline EEG data were processed using MNE-Python^54^ as well as custom scripts written for Python 3.8. First, to remove slow baseline drifts and high frequency noise, EEG data were filtered with a one-pass, zero-phase, non-causal finite impulse response bandpass filter (parameters: Hamming window with 0.0194 passband ripple and 53 dB stopband attenuation; filter length: 8449 samples). The lower bandpass edge was set at 0.10 Hz (transition bandwidth: 0.10 Hz; −6 dB cut-off frequency: 0.05 Hz) and the upper bandpass edge at 80 Hz (transition bandwidth: 20 Hz; −6 dB cut-off frequency: 90.00 Hz). Additionally, to attenuate artefacts caused by line noise, a notch filter was applied to the data (lower passband edge: 49.38 Hz, upper passband edge: 50.52 Hz, transition bandwidth: 0.50 Hz). Second, stereotypical signal artefacts caused by ocular movements were corrected using a regression algorithm as proposed by Gratton *et al*.^55^ Third, power spectral density (PSD) estimates by calculating the periodogram via discrete Fourier transform (constraint to 2.0 to 20 Hz at 0.5 Hz resolution Hamming window) for a sliding window of 2 seconds in the time dimension, 0 to +30 seconds around the start of the regulation phase in each training trial and then averaging over the 2 seconds segments. This was done for each channel and training trial individually.^56^ The power spectral density (PSD, μV^2^/Hz) for the frequency bands was computed by summing up the PSD estimates for each individual frequency within a specific frequency band (e.g. delta = PSD 2.0 Hz + PSD 2.5 Hz + PSD 3.0 Hz + PSD 3.5 Hz, etc.). Finally, for each participant, the frequency band PSD estimates were transformed to decibel (dB) units by means of logarithmic transformation (i.e. delta_dB = 10 × log10(delta)) and averaged across trails for better comparison across frequency bands.

#### Analyses of EEG measures

To assess whether increased neurofeedback training time led to changes in PSD in the trained frequency bands, time (i.e. session number) was treated as continuous variable with observations at session 1 through 10. The two-level mixed effects model included group (between-subjects categorical predictor; ADR-NF versus BTR-NF), time and the group by time interaction as fixed effects. Furthermore, participants’ age, gender, CEQ and THI at baseline were introduced as categorical covariates. As described above, all models estimated by-subjects random effects and were fitted by restricted log likelihood estimation.

## Results

### Primary outcomes

**Figure 3:**
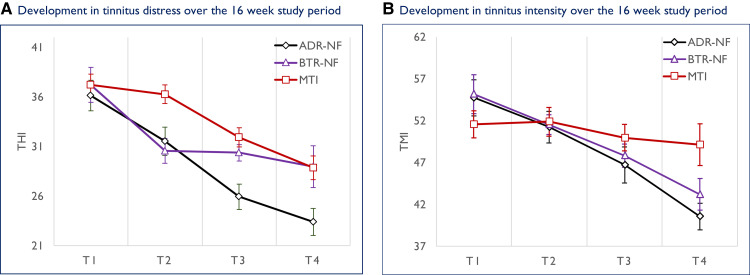
**Descriptive statistic for the two primary outcomes**. Figure 3 shows the development in tinnitus distress (left panel) and in tinnitus intensity (right panel) for ADR-NF, BTR-NF and MTI, respectively. On both outcomes, there is a trend for greater improvement in ADR-NF. Data point represents group means. Error bars, corrected according to Morey,^57^ depict 95% confidence intervals. THI, Tinnitus Handicap Inventory; TMI, Tinnitus Magnitude Index.

Figure 3 shows the descriptive statistic (means and confidence intervals) of THI (A) and TMI (B), respectively, for the ITT sample. Most salient is the superior response for ADR-NF on the THI, where the reduction in tinnitus distress between pre- (T_1_, THI = 36.13) and post-interventions (T_3_, THI = 25.94) constitutes a clinically relevant change.^58^

### Tinnitus Handicap Inventory (THI)

In the intention-to-treat analysis (ITT, *n* = 94), repeated measures mixed models showed a significant main effect of time (weeks) on THI scores at T_4_, (with BTR-NF as reference for the intervention: estimate: −7.66, SE: 2.46, *P* = 0.002; with MTI as reference for the intervention: estimate: −6.20, SE: 2.22, *P* = 0.006). That is, both BTR-NF and MTI reported highly significant benefit of the interventions over time, in terms of tinnitus distress reduction. Concerning hypothesis 1, interaction analyses showed that ADR-NF had lower THI scores compared to BTR-NF, both at T_3_ (ADR-NF: estimate: −3.51, SE: 3.34, *P* = 0.29) and at T_4_ (ADR-NF: estimate: −4.63, SE: 3.37, *P* = 0.17). Table 2 (upper panel) shows all time × group interactions for THI. Despite the trend in the expected direction, none of the time comparisons between BTR-NF and ADR-NF were significant. Similarly, between BTR-NF and MTI, there were no significant interactions. The comparisons between MTI and ADR-NF did show trends towards significance, at both T_3_ (ADR-NF: estimate: −6.08, SE: 3.18, *P* = 0.06) and T_4_ (ADR-NF: estimate: −6.09, SE: 3.21, *P* = 0.06). Taken together, ADR-NF showed the most marked reduction in tinnitus distress, followed by BTR-NF and MTI.

**Table 2:** Interactions for the Tinnitus Handicap Inventory (upper panel) and Tinnitus Magnitude Index (lower panel) for ITT

	BTR-NF versus ARD-NF			MTI versus BTR-NF			MTI versus ADR-NF		
Time	EST.	SE	*P*	EST.	SE	*P*	EST.	SE	*P*
Interactions across all levels of comparisons for the Tinnitus Handicap Inventory									
T_2_	−0.44	2.84	0.88	−4.71	2.82	0.10	−4.27	2.71	0.12
T_3_	−3.51	3.34	0.29	−2.57	3.28	0.43	−6.08	3.18	0.06
T_4_	−4.63	3.37	0.17	−1.46	3.30	0.66	−6.09	3.21	0.06
Interactions across all levels of comparisons for the Tinnitus Magnitude Index									
T_2_	−2.04	3.89	0.60	−2.59	3.85	0.50	−4.63	3.70	0.21
T_3_	−1.40	4.27	0.74	−5.66	4.20	0.18	−7.09	4.08	0.08
T_4_	−4.19	4.14	0.31	−8.14	4.10	<0.05	−12.33	3.95	<0.01

Table 2 shows the interactions of primary interest in the study. Top panel shows interactions for the Tinnitus Handicap Inventory. Lower panel shows interactions for the Tinnitus Magnitude Index across all levels of comparisons. Numbers before the comparison (e.g. 2.1 BTR-NF versus ADR-NF) correspond to numbers referred to in the manuscript.

ITT, intention-to-treat; EST, estimate based on imputations; SE, standard error of estimate; *P*, *P*-value; T_2_, mid-point assessment; T_3_, post-intervention assessment; T_4_, follow-up (3 months); ADR-NF, alpha/delta ratio neurofeedback; BTR-NF, beta/theta neurofeedback; MTI, minimal treatment intervention.

When performing the per-protocol analysis (PP, *n* = 78), results mirrored those of the ITT analyses. Notably, there was closer approximation between the two neurofeedback interventions at T_4_ compared to the ITT analysis (ADR-NF: estimate: −1.84, SE: 2.69, *P* = 0.49, see Supplementary Table 3 for all ITT and PP time, group and time × group interactions).

### Tinnitus Magnitude Index (TMI)

None of the analyses for the TMI with MTI and BTR-NF as reference found significant main effects of time. Thus, in contrast to THI, participants in neither BTR-NF nor MTI reported beneficial effects of the intervention, related to the perceived intensity of tinnitus.

Table 2 (lower panel) shows time × group comparisons for TMI. As with THI, ADR-NF reported the most benefit in terms of reduced tinnitus intensity compared to BTR-NF, T_4_ (ADR-NF: estimate: −4.19, SE: 4.14, *P* = 0.31) and to MTI, T_4_ (ADR-NF: estimate: −12.33, SE: 3.95, *P* = 0.01). Still, concerning hypothesis 1, results showed no significant interaction effects between ADR-NF and BTR-NF. For hypothesis 2, results did show significant interactions: at follow-up (T_4_), BTR-NF participants showed marginally significant improvement compared to MTI (BTR-NF: estimate: −8.14, SE: 4.10, *P* = 0.048). Similarly, the contrast between ADR-NF and MTI was significant at follow-up (T_4_, ADR-NF: estimate: −12.33, SE: 3.95, *P* = 0.002).

For the PP sample, the pattern of results deviated somewhat from the ITT analyses, with differences between MTI and BTR-NF being more pronounced at both post-intervention (T_3_, BTR-NF: estimate: −8.10, SE: 3.82, *P* = 0.04) and follow-up (T_4_, BTR-NF: estimate: −9.13, SE: 4.09, *P* = 0.03). Post-intervention (T_3_) and follow-up (T_4_), differences between MTI and ADR-NF were statistically significant, with ADR-NF reporting lower tinnitus intensity scores. None of the interactions between ADR-NF and BTR-NF reached statistical significance, despite ADR-NF being the most beneficial treatment (see Supplementary Table 4 for all ITT/PP time, group and time × group interactions).

### Secondary and exploratory outcomes

The ITT and PP analyses of secondary outcomes largely mirrored those of the primary outcomes. For the TFI, there were significant interactions across the levels of measurement between MTI and ADR-NF in both the ITT and PP analyses. For the ITT analysis, results showed lower tinnitus distress for ADR-NF at both post-intervention (T_3_) and follow-up (T_4_), (T_3_, estimate: −10.74, SE: 3.49, *P* = 0.002; T_4_, estimate: −9.77, SE: 3.63, *P* = 0.008). As with the two primary outcomes, ADR-NF reported the greatest benefit of treatment over the course of the study period, but differences between ADR-NF and BTR-NF remained insignificant (see Supplementary Table 5).

Insomnia severity, measured with the ISI, showed a significant main effect of time at post-intervention (T_3_) and an effect trending towards significance at follow-up (T_4_) for the MTI group (T_3_, estimate: −1.93, SE: 0.72, *P* = 0.008; T_4_, estimate: −1.47, SE: 0.77, *P* = 0.06) in the ITT analysis. None of the interactions between groups were significant across levels of measurement (for results of both ITT and PP analyses, see Supplementary Table 6).

Results of the ITT analysis for depression, measured with the PHQ, found a significant effect of time both post-intervention (T_3_, BTR-NF: estimate: −1.18, SE: 2.06, *P* = 0.04, MTI: estimate: −1.44, SE: 0.52, *P* = 0.006) and follow-up (T_4_, BTR-NF: estimate: −1.29, SE: 0.62, *P* = 0.04, MTI: estimate: −1.33, SE: 0.57, *P* = 0.02). None of the interactions reached statistical significance (see Supplementary Table 7 for all ITT and PP analyses).

For treatment credibility and expectations (CEQ), the ITT analyses showed a significant effect of time for both BTR-NF and MTI (T_3_, BTR-NF: estimate: −2.82, SE: 1.39, *P* = 0.04, MTI: estimate: −3.56, SE: 1.30, *P* = 0.007). That is, both BTR-NF and MTI participants reported a decrease in treatment credibility and expectations over the course of treatment. Regarding interactions, none of them reached statistical significance (see Supplementary Table 8 for results of both ITT and PP analyses).

### EEG data

**Figure 4:**
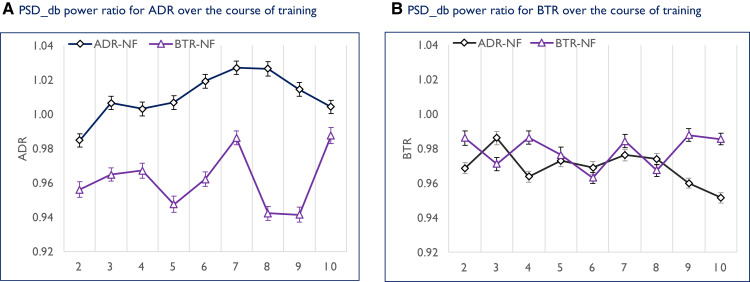
**Descriptive statistic—EEG-frequency development trajectory over the course of training**. Descriptive statistic showing the mean alpha/delta ratio [left panel, (**A**)] and beta/theta ratio [right panel, (**B**)] for each training session. [Left panel, (**A**)] The alpha to delta ratio was consistently elevated in ADR-NF compared to BTR-NF over the training period. [Right panel, (**B**)] Comparable beta/theta ratios were produced between the groups over the course of training. Note: Each data point represents the average EEG-frequency ratio in PSD_dB for each of the nine individual sessions. *x*-Axis = Session no. *y*-Axis = PSD_dB ratio. Error bars = 95% confidence intervals corrected according to Morey^57^.

Based on observed outcome, Fig. 4 shows the mean ratio [power spectral density decibel (PSD_dB)] across sessions 2 to 10, for the alpha to delta (ADR) and beta to theta (BTR) frequency bands. A consistent pattern of higher ADR for ADR-NF compared to BTR-NF is shown in the left panel (A). This suggests that the alpha to delta ratio protocol established the desired EEG frequency pattern in ADR-NF, and not in BTR-NF. Conversely, no clear pattern of superiority was evident for the BTR protocol (right panel, B). As seen, a mixed pattern emerged, with near identical BTR over the course of training between ADR-NF and BTR-NF.

For the ADR, a repeated measures mixed model regression was performed with PSD_dB as the outcome variable, session and group as predictor variables. The repeated measures mixed regression showed a highly significant main effect of session on ADR enhancement [*F*(8,389) = 7.14, *P* < 0.001], no main effect of group [*F*(1,46) = 1.26, *P* = 0.30] but a significant session × group interaction [*F*(8,389) = 11.61, *P* < 0.001]. As shown in Table 3 (left panel), a between-group contrast analysis (group × session) showed no differences at any level of comparison.

**Table 3:** Between-group × session contrast analyses for ADR and BTR

	Group × session contrasts—ADR					Group × session contrasts—BTR				
	EST					EST				
Session	ADR-NF	BTR-NF	Diff	SE	*P*	ADR-NF	BTR-NF	Diff	SE	*P*
Session 2	**0.98**	0.95	0.03	0.05	0.51	0.98	0.99	−0.01	0.04	0.86
Session 3	**1.01**	0.95	0.06	0.05	0.25	**1.00**	0.96	0.04	0.04	0.37
Session 4	**1.00**	0.97	0.03	0.05	0.56	0.97	0.97	0.00	0.04	0.93
Session 5	**1.02**	0.94	0.07	0.05	0.14	**0.98**	0.96	0.01	0.04	0.76
Session 6	**1.01**	0.95	0.06	0.05	0.23	**0.98**	0.95	0.03	0.04	0.53
Session 7	**1.03**	0.97	0.05	0.05	0.27	**0.99**	0.98	0.01	0.04	0.79
Session 8	**1.02**	0.94	0.08	0.05	0.09	**0.98**	0.96	0.02	0.04	0.64
Session 9	**1.00**	0.95	0.05	0.05	0.30	0.96	0.97	−0.01	0.04	0.86
Session 10	**1.00**	0.97	0.03	0.05	0.57	0.95	0.96	0.00	0.04	0.93

Table 3 contrast analysis group × session analyses for both ADR and BTR. (Left panel) Based on estimated marginal means, there appears to be a tendency for better ADR enhancement in ADR-NF compared to BTR-NF across all levels of comparison. (Right panel) The analysis shows a near identical BTR enhancement between ADR-NF and BTR-NF across sessions. In bold = comparisons where ADR-NF > BTR-NF.

EST, estimated marginal means; SE, standard error; Diff, difference between ADR-NF and BTR-NF.

For BTR, the repeated measures mixed regression showed a highly significant main effect of session on BTR enhancement [*F*(8,385) = 8.82, *P* < 0.001], no main effect of group [*F*(1,47) = 0.06, *P* = 0.81] but a significant session × group interaction [*F*(8,385) = 10.61, *P* < 0.001]. The group × session contrast analysis found no differences at any level of comparison between BTR-NF and ADR-NF (Table 3, right panel).

With session two as reference, Table 4 shows the session × group comparisons for ADR (far-left and middle-left panels) and BTR (middle-right and far-right panels) for ADR-NF and BTR-NF, respectively. For ADR, the positive PSD_dB values in all comparisons for ADR-NF (far-left panel) mean that participants in this intervention managed to enhance the ADR consistently throughout the course of the training period, at least compared to session two. Moreover, *t*-tests showed this enhancement to be significantly higher in five of eight comparisons. In contrast, no such pattern was established for BTR-NF (middle-left panel). The change in PSD_dB was positive in only four of eight comparisons, and none were statistically significant (middle-left panel).

**Table 4:** Session × group comparisons

	Change (Δ) in ADR—session 2 as baseline						Change (Δ) in BTR—session 2 as baseline					
	ADR-NF			BTR-NF			BTR-NF			ADR-NF		
Session	Δ PSD_dB	Δ %	*P*	Δ PSD_dB	Δ %	*P*	Δ PSD_dB	Δ %	*P*	Δ PSD_dB	Δ %	*P*
Session 2	+0.98			+0.95			+0.99			+0.98		
3 versus 2	**+0**.**022**	**+2**.**26**	**<0.001**	−0.001	−0.07	ns	**−0**.**025**	**−2**.**57**	**<0.001**	**+0**.**017**	**+1**.**75**	**<0.01**
4 versus 2	+0.013	+1.27	ns	+0.014	+1.48	ns	**−0**.**016**	**−1**.**59**	**<0.05**	−0.010	−1.03	ns
5 versus 2	**+0**.**034**	**+3**.**43**	**<0.001**	−0.009	−0.88	ns	**−0**.**023**	**−2**.**37**	**<0.01**	−0.003	−0.28	ns
6 versus 2	**+0**.**027**	**+2**.**72**	**<0.05**	−0.001	−0.14	ns	**−0**.**037**	**−3**.**73**	**<0.005**	−0.003	−0.31	ns
7 versus 2	**+0**.**045**	**+4**.**51**	**<0.001**	+0.021	+2.25	*ns*	−0.009	−0.92	*ns*	+0.011	+1.08	ns
8 versus 2	**+0**.**039**	**+3**.**97**	**<0.05**	−0.010	−1.04	*ns*	−0.028	−2.87	*ns*	−0.002	−0.18	ns
9 versus 2	+0.021	+2.12	ns	+0.005	+0.47	*ns*	−0.022	−2.19	*ns*	−0.019	−1.93	ns
10 versus 2	+0.015	+1.48	ns	+0.012	+1.91	*ns*	−0.031	−3.14	*ns*	−0.025	−2.53	ns

Table 4 shows the EEG spectral change in PSD_dB, percentage change (%) and significance level (*t*-test) for ADR-NF and BTR-NF respectively, when compared to session two. Far-left panel shows that ADR-NF managed to enhance the ADR in each comparison to session two. This indicates that the protocol seemed to produce the desired effect throughout the intervention period for ADR-NF. (Middle-left panel) For BTR-NF, no such clear pattern of ADR enhancement was achieved, with some sessions showing enhancement and some attenuation compared to session two. Middle-right panel shows that the BTR protocol did not achieve the intended augmentation of the BTR for BTR-NF. In fact, all eight comparisons with session two showed the opposite pattern than was expected for BTR-NF: an attenuation of the BTR. (Far-right panel) The same trend for ADR-NF with most sessions showing a decrease in the BTR when compared to session two. Δ = change. NB! *P*-values are based on *t*-tests and adjusted for familywise error rates. The comparisons in bold indicate significant differences in ratio change compared to session 2.

For BTR, the negative PSD_dB value across all comparisons for BTR-NF (middle-right panel) indicates that compared to session two, all subsequent sessions produced lower BTR. Moreover, the EEG oscillatory response was significantly attenuated in four of eight comparisons. For ADR-NF (far-right panel), the results were like those in BTR-NF, but with two exceptions. The BTR response was enhanced in session three versus session two, and again in session seven versus session two, respectively.

To summarize: as evident from Fig. 4 (far-left panel), the ADR protocol established the desired EEG frequency pattern for ADR-NF. Conversely, the BTR protocol seemed to affect oscillatory activity patterns opposite to what was intended for BTR-NF (middle-right panel). That is, compared to session two, all eight comparisons showed upregulation of theta EEG activity and downregulation of beta EEG activity.

## Discussion

This randomized controlled trial compared 10 sessions of ADR-NF with a neurofeedback control condition (BTR-NF) and a non-neurofeedback MTI.

### Specificity of the ADR-NF protocol in alleviating tinnitus distress and intensity (hypothesis 1)

Our main research question was to further clarify whether reduced tinnitus distress, measured with the THI, and tinnitus intensity (measured with the TMI), were related to ADR-NF specificity. Despite a trend in the expected direction, the ITT analyses showed that there were no superior effects of ADR-NF on any of the two primary outcomes, when results were compared to BTR-NF. These findings were moreover supported by results from the per-protocol (PP) analyses; there was no evidence of specificity, although the trend in the data remained.

### An association between observed trend and ADR-EEG activity

The results of the EEG analyses showed no difference between ADR-NF and BTR-NF on the ADR during training, at least when compared session by session (Table 3). Nonetheless, the session × group interaction indicated marked differences in the ADR over the course of the training period. More specifically, data showed that the ADR was elevated for ADR-NF compared to BTR-NF (Fig. 4A). In addition, when using session two as reference, ADR-NF showed greater indices of successful regulation of the ADR (Table 4). Combining this finding with those of previous studies that used the ADR protocol suggests that the protocol worked as intended. Both Dohrmann *et al*.^15^ and Crocetti *et al*.^16^ found a similar tendency for ADR increase during training. More compelling however is the study by Güntensperger *et al*.^17^: they compared pre- and post-intervention resting-state EEG activity, and they too found an increase in the ADR.

Since in our study, all variables, except neural targets, were comparable between ADR-NF and BTR-NF, it is worth considering parameters which, if they were different, might have augmented the difference between ADR-NF and BTR-NF on the two primary outcome measures.

### Factors that might have augmented treatment response in ADR-NF

Participants in the present study had 10 neurofeedback sessions. Although this number of sessions might be adequate for young healthy adults, older adults might require additional training sessions, to benefit from the training.^59^ The sample in the present study was middle-aged, and therefore, 10 sessions of neurofeedback might not have been sufficient, to achieve a full therapeutic response. Moreover, it has been proposed that neuroplastic changes, initiated during neurofeedback therapy, continue to develop in the ensuing months.^60^ For this reason, the post-intervention and follow-up time points in our study might have been too close in proximity, to fully capture the long-term benefits initiated during training in ADR-NF. Lastly, it may well be an over-simplification to view individuals with tinnitus as one homogenous population with the same, distinct neuronal activity pattern. For example, since the inception of the SLIM model, more recent research has shown that resting-state EEG fluctuates to a considerable degree inter-individually in tinnitus populations.^29,61^ Therefore, a one-size fits all treatment, such as ADR-NF, might be too unspecific for most individuals with tinnitus, specifically those whose EEG activity patterns do not conform to those suggested by the SLIM.

Nonetheless, these questions might be superfluous, if one adopts the view, that the difference between ADR-NF and BTR-NF was due to chance, or alternatively not related to the ADR-NF protocol at all.

### Why the trend in the primary outcome data might not be related to ADR-NF protocol parameters

Experimenters who are not blind to treatment risk influencing participants in a direction that confirms a hypothesis.^62^ This bias might have been a factor in the present study, where experimenters could have sought to influence ADR-NF participants, albeit subconsciously, to be more accepting and positive towards the treatment. Second, there is the issue of spatial specificity raised by Güntensperger *et al*.^25^ In short, the central question is whether frontocentral electrodes, as used by the ADR-NF protocol, are ideally located for capturing EEG activity, which stems from the temporal lobes. Although projecting forwards to frontocentral regions,^15^ the activity from PAC might be blended with brainwave activity, which originates from other brain regions. Although these issues cannot be resolved in the present study, it is worth keeping the spatial specificity observation in mind, when interpreting the data from the present study.

### Impact of neurofeedback non-specific effects on treatment outcome (hypothesis 2)

#### The moderating influence of control beliefs

The second objective of the present study was to help further our understanding, of the mechanisms contributing to neurofeedback treatment efficacy. The analyses found no difference between ADR-NF/BTR-NF versus MTI on any outcome measures, when assessed upon intervention completion (T_3_). In other words, there seemed to be no added effect from neurofeedback non-specific factors. The only difference that emerged on the primary outcome measures was on the TMI. At 3-months follow-up (T_4_), both neurofeedback groups reported significantly lowered tinnitus intensity compared to MTI. Since the TMI had high reliability, when assessed at the four time points, we assume that the differential outcome was not the result of an instable measurement instrument. At this stage, we can only speculate why the positive development trajectory continued on the TMI for both ADR-NF and BTR-NF in the ensuing weeks. Previous studies have shown that neurofeedback activates neural correlates of cognitive control, which is independent of the feedback signal^63^ and moreover, can lead to an overestimation of one’s control beliefs.^32^ In the context of tinnitus management, control beliefs are considered particularly effective.^64^ Therefore, the reduced tinnitus intensity over time, observed in the two neurofeedback conditions, might at least in part be moderated by augmented control beliefs.

### The absence of neuroenhancement

Given the debate in the literature concerning neurofeedback non-specific factors (e.g. Schönenberg *et al*.^32^ and Thibault *et al*.^30^), it is rather surprising that treatment credibility and expectations did not differ between the two neurofeedback groups and MTI. We offer three explanations for this none-difference between intervention arms: first, the therapeutic elements in MTI might have had sufficient appeal to offset any additional treatment expectations in the two neurofeedback conditions. Second, tinnitus patients have often tried several treatment options to no avail.^65^ Therefore, we concur with Güntensperger *et al*.,^17^ specifically that this population may be less expectant, when evaluating new treatment options. Third, the pre-intervention information (see study protocol^34^) stated that we wanted to compare two neurofeedback treatments and importantly, whether it mattered, what frequency bands were trained. In doing so, we might inadvertently have lowered treatment expectations in both groups. It is also noteworthy that all groups reported a reduction on the CEQ over the course of the intervention period. This suggests that the perceived treatment credibility and expectations did not exert a significant impact on outcomes.

### The paradox of the BTR protocol

An interesting observation from the analysis of BTR-EEG data was the apparent absence of an effect, or at least one in the expected direction. In fact, the opposite seemed to happen, with a BTR decrease, and not increase, in the BTR-NF group over the course of the intervention period (Table 4). This tendency is by no means easy to account for. Nonetheless, examining the reported strategies used during training might provide a clue (see Supplementary Fig. 1). When BTR-NF participants were asked to report which mental strategies they primarily used during training, around half of them stated that they used mental imagery. Since theta activity correlates with a state of internally directed attention,^66^ and beta activity with more externally driven attentional states (e.g. coping with behavioural demands^67^), then engaging in mental imagery might be more closely related to the former. In short, BTR-NF participants might have used mental strategies that induced theta, and not beta EEG activity.

### Specificity of ADR-NF on tinnitus distress, insomnia and symptoms of depression (secondary outcomes)

The results of the secondary outcomes confirm the conclusions drawn from the primary outcomes. That is, there was no evidence to suggest specificity of ADR-NF in the lower levels of insomnia (ISI), depression (PHQ-9) and tinnitus distress (TFI) reported neither at post-intervention (T_3_) nor follow-up (T_4_). Significant interactions did occur between ADR-NF and MTI at all levels of measurement on the TFI. Since this difference was absent in the comparisons between BTR-NF and MTI, we speculate whether this was the result of an experimenter bias. As suggested earlier, the experimenter bias could have influenced the behaviour of researchers involved with ADR-NF and could have had a carry-over effect to ADR-NF participants. This of course remains purely speculative at this stage and needs further confirmatory studies.

### Limitations

First, although larger than previous studies, the sample size in the present study is too small to detect a small effect size (i.e. Cohen’s *d* = 0.2). Assuming that the effect of ADR-NF is not medium (as was the case in the a priori sample size calculation, cf., study protocol^34^) but small, the study would have needed to include at least 174 participants. This emphasizes one main challenge of conducting neurofeedback studies: they are in nature very time intensive, both for researchers and participants. Such large-scale studies require a multi-site set-up with several collaborators involved. Because the size of our sample is only near adequate to detect a medium effect size, any conclusions must be considered tentative in nature. Another limitation is that a large amount of EEG data had to be removed from the analysis due to outliers. Unfortunately, this is not an unusual phenomenon in neurofeedback studies.^29,68^ Yet, given that the study was already underpowered, the exclusion of data limits the generalizability of the findings. In addition, similar to Dohrmann *et al*.^15^ and Crocetti *et al*.,^16^ no pre- and post-intervention EEG resting-state data were collected. Therefore, we cannot address the question, whether the training protocols led to neuroplastic changes and importantly, how these might have related to long-term outcomes. However, since studies have shown that resting-state EEG might not be a very reliable indicator of neuroplastic changes,^68^ it remains uncertain whether this is in fact a true limitation. We also consider it a limitation to have used the TMI to track progress in the reduction in tinnitus intensity. Nonetheless, the decision was made to include it, because to date, no other reliable and objective way of measuring tinnitus intensity and/or loudness has been developed.^44^ Because of this decision, the hypothesized association between symptom control and reduced tinnitus intensity discussed earlier rests on findings from a not widely used research instrument. Finally, missingness present in outcome data is a limitation, as the analysis for the ITT population is not possible when outcome data are missing. Therefore, the analysis had to include imputation, which may lead to less certain values. However, the ITT analysis did not deviate from the analysis on the PP population.

## Conclusion and future directions

The primary goal of this randomized controlled trial was to address the question whether there is specificity for the ADR-NF training protocol. Although no significant benefit of ADR-NF over BTR-NF was found, the trend in the primary outcome makes future explorations of the ADR-NF protocol worth considering. An obvious adjustable parameter would be increasing the number of training sessions or alternatively, explore whether treatment effects are better in younger adults. Despite requiring considerable investments, it would also be interesting, and a step in the direction towards greater specificity, to tailor the therapy protocol to individual resting-state EEG oscillatory patterns. A first attempt at this was already made by Güntensperger *et al*.,^17^ who could report beneficial effects of an individualized alpha peak protocol. Besides these more pertinent and critical issues, it would however also be of interest to manipulate control beliefs, in order to further elucidate the influence of this hypothesized non-specific factor.

Regarding the second aim of this study, i.e. the examination of neurofeedback non-specific factors, a surprising discovery was that assumingly, the prospect of entering neurofeedback training did not result in heightened treatment expectations. This shows that neuromodulation by its very nature might not coax people into having blind faith in the capacity to bring about positive health benefits.

On a final note, one might ask, as does Hammond,^69^ whether it matters that the majority of treatment benefits in neurofeedback may stem from a placebo response, as long as the magnitude of improvement is similar to well-established treatments? For example, compared to psychotherapy, neurofeedback might appeal more to certain individuals, not least those who are convinced that neurofeedback targets the root cause of their symptoms, i.e. aberrant brain activity. We therefore suggest that it might be too premature to discard the idea of neurofeedback in the treatment of chronic tinnitus.

## Supplementary Material

fcad185_Supplementary_Data

## Data Availability

The data that support the findings of this study are openly available in figshare at https://doi.org/10.6084/m9.figshare.21500979.

## Abbreviations

ADR: alpha to delta ratio
ADR-NF: alpha/delta ratio neurofeedback
AIC: Akaike’s Information Criterion
BTR-NF: beta/theta ratio neurofeedback
CBT: Cognitive Behavioural Therapy
CEQ: Credibility and Expectancy Questionnaire
dB: decibel
EEG-NF: EEG neurofeedback
ISI: Insomnia Severity Index
ITT: intention-to-treat
MTI: minimal treatment intervention
PHQ-9: Patient Health Questionnaire-9
PP: per protocol
PSD: power spectral density
SLIM: synchronization by loss of inhibition model
TFI: Tinnitus Functional Index
THI: Tinnitus Handicap Inventory
TMI: Tinnitus Magnitude Index
