# Supplementary material for: Does it matter what is trained? A randomized controlled trial evaluating the specificity of alpha/delta ratio neurofeedback in reducing tinnitus symptoms
Source: Brain Commun. 2023 Jun 26;5(4):fcad185. doi: 10.1093/braincomms/fcad185 (PMC10481778; doi:10.1093/braincomms/fcad185)
Supplement: fcad185_Supplementary_Data [file fcad185_Supplementary_Data.pdf]

Supplementary Table 1 Audiological sample characteristics for ITT

	Total N	ADR-NF		BTR-NF		MTI			
		Mean	SD	Mean	SD	Mean	SD	F	*p*
**Tinnitus Characteristics**									
PTA 8kHz left ear	90	19	16	20	16	19	14	0,04	0,96
PTA 8kHz right ear	90	17	12	19	16	18	13	0,28	0,76
PTA 12.5 kHz left ear	73	24	13	18	14	25	17	1,45	0,24
PTA 12.5 kHz right ear	73	23	11	17	15	24	16	1,37	0,26
Tinnitus pitch match kHz	92	6,8	4,1	7,1	2,9	5,9	3,5	0,20	0,82
Loudness Discomfort level dB	92	94	14	92	12	92	14	0,91	0,41

Supplementary table1 shows tinnitus characteristics for the Intent-to-treat (ITT) sample, including F statistics. The groups did not differ significantly on any of the variables at pre-intervention (T1). SD = Standard Deviation. PTA = pure tone average, dB = decibel. PTA 8kHz = average (500 hZ, 1kHz, 2 kHz, 3 kHz, 4 kHz, 6 kHz, 8 kHz). 12 kHz = average (500 Hz, 1kHz, 2 kHz, 3 kHz, 4 kHz, 6 kHz, 8 kHz, 9kHz, 10 kHz, 12 kHz).

Supplementary table 2 Inclusion- and exclusion criteria

**Inclusion**	**Exclusion**
• Age + 18 years	• Objective tinnitus, either vascular or non-vascular in origin
• Chronic, subjective tinnitus with a duration of ≥ 6 months	• Acute inflammatory disease of the ear/ears
• Mild tinnitus distress, THI ≥ 18	• Conductive hearing loss
• Signed informed consent	• Blockage of the ear canal by, for example, cerumen, where removal is not desired by the individual
	• Any current treatment for tinnitus
	• Severe mental health issues (moderate/severe depression, bipolar disorder, psychosis)
	• ADHD
	• Current use of a psychotropic drug for a mental health condition
	• Substance abuse
	• Previous or current neurofeedback treatment
	• Previous or current neurological conditions (e.g., a history of seizures, brain tumor, hemorrhage/ stroke)

Supplementary table 2 shows the inclusion- and exclusion criteria for the study. The information can also be found in the study protocol[34].

Supplementary table 3 Tinnitus Handicap Inventory parameters in both ITT and PP samples

	MTI as reference group						BTR-NF as reference group					
	ITT			PP			ITT			PP		
	**EST.**	**SE**	***P***	**EST.**	**SE**	***P***	**EST.**	**SE**	***P***	**EST.**	**SE**	***P***
**Time**												
T2	-0,79	1,88	0,67	-1,11	1,70	0,51	-5,50	2,09	**< 0.01**	-6,28	1,96	**< 0.01**
T3	-4,12	2,20	**0,06**	-3,72	1,64	**< 0.05**	-6,69	2,43	**< 0.01**	-8,26	1,95	**< 0.001**
T4	-6,20	2,22	**< 0.01**	-6,22	1,73	**< 0.001**	-7,66	2,46	**< 0.01**	-9,25	2,02	**< 0.001**
**Group**												
MTI vs. ADR-NF	0,72	2,15	0,74	-0,06	2,23	0,99						
MTI vs BTR-NF	0,51	2,20	0,82	0,82	2,40	0,73						
BTR-NF vs. ADR-NF							0,21	2,26	0,93	-0,88	2,44	0,72
**Time x Group**												
MTI vs. ADR-NF												
T2	-4,27	2,71	0,12	-2,81	2,48	0,26						
T3	-6,08	3,18	**0,06**	-3,99	2,36	0,09						
T4	-6,09	3,21	**0,06**	-4,87	2,48	**0,051**						
MTI vs BTR-NF												
T2	-4,71	2,82	0,10	-5,17	2,60	**< 0.05**						
T3	-2,57	3,28	0,43	-4,54	2,55	0,08						
T4	-1,46	3,30	0,66	-3,03	2,66	0,26						
BTR-NF vs. ADR-NF												
T2							-0,44	2,84	0,88	2,36	2,66	0,38
T3							-3,51	3,34	0,29	0,55	2,58	0,83
T4							-4,63	3,37	0,17	-1,84	2,69	0,49

Supplementary table 3 Data from regression models showing all time, group and time ^{X} group interactions for both ITT and PP samples with the Tinnitus Handicap Inventory as outcome variable. Data are provided with both MTI and BTR-NF respectively as reference groups. Since multiple imputations were used, the model include estimates (EST.), standard error (SE) and p values.

Supplementary table 4 TMI parameters in both ITT and PP samples with both MTI and BTR-NF as reference groups

	MTI as reference group						BTR-NF as reference group					
	ITT			PP			ITT			PP		
	EST.	SE	*P*	EST.	SE	*P*	EST.	SE	*P*	EST.	SE	*P*
**Time**												
T2	0,55	2,56	0,83	0,96	2,47	0,70	-2,04	2,85	0,48	-6,28	1,96	0,35
T3	-0,92	2,84	0,75	-0,37	2,45	0,88	-6,61	3,11	0,34	-8,26	1,95	**< 0.01**
T4	-2,02	2,76	0,47	-1,68	2,63	0,52	-10,16	3,03	0,88	-9,25	2,02	**< 0.001**
**Group**												
MTI vs. ADR-NF	2,99	4,24	0,48	3,58	4,45	0,43						
MTI vs BTR-NF	3,05	4,32	0,48	2,90	4,78	0,55						
BTR-NF vs. ADR-NF							-0,06	4,43	0,99	0,68	4,86	0,89
**Time x Group**												
MTI vs. ADR-NF												
T2	-4,63	3,70	0,21	-3,85	3,65	0,29						
T3	-7,09	4,08	0,08	-8,63	3,52	**< 0.05**						
T4	-12,33	3,95	**< 0.01**	-12,02	3,78	**< 0.01**						
MTI vs. BTR-NF												
T2	-2,59	3,85	0,50	-3,71	3,82	0,33						
T3	-5,66	4,20	0,18	-8,10	3,82	**< 0.05**						
T4	-8,14	4,10	**< 0.05**	-9,13	4,09	**< 0.05**						
BTR-NF vs. ADR-NF												
T2							-2,04	3,89	0,60	-0,15	3,96	0,97
T3							-1,40	4,27	0,74	-0,53	3,87	0,89
T4							-4,19	4,14	0,31	-2,89	4,15	0,49

Supplementary table 4 Data from regression models for both ITT and PP samples. Data are provided with both MTI and BTR-NF respectively as reference groups. Since multiple imputations were used, the model include estimates (EST.), standard error (SE) and p values.

Supplementary table 5 Interactions for the Tinnitus Functional Index

	MTI as reference group						BTR-NF as reference group					
	ITT			PP			ITT			PP		
	**EST.**	**SE**	***P***	**EST.**	**SE**	***P***	**EST.**	**SE**	***P***	**EST.**	**SE**	***P***
**TFI**												
**Time**												
T2	-1,50	1,95	0,44	-0,68	2,06	0,74	-4,77	2,13	0,03	-6,01	2,42	**< 0.05**
T3	-3,55	2,43	0,15	-2,17	2,01	0,28	-8,53	2,66	**< 0.01**	-10,05	2,49	**< 0.001**
T4	-4,92	2,55	0,06	-3,43	2,12	0,11	-9,26	2,75	**< 0.001**	-10,01	2,47	**< 0.001**
**Time x Group**												
MTI vs. ADR-NF												
T2	-7,90	2,76	**< 0.01**	-7,53	3,06	**< 0.05**						
T3	-10,74	3,49	**< 0.01**	-9,82	3,00	**< 0.01**						
T4	-9,77	3,63	**< 0.01**	-9,89	3,07	**< 0.01**						
MTI vs. BTR-NF												
T2	-3,27	2,91	0,26	-5,33	3,17	0,09						
T3	-4,99	3,61	0,17	-7,88	3,20	< 0.05						
T4	-4,35	3,75	0,25	-6,58	3,25	< 0.05						
BTR-NF vs. ADR-NF												
T2							-4,63	2,86	0,11	-2,20	3,31	0,51
T3							-5,75	3,65	0,12	-1,94	3,34	0,56
T4							-5,42	3,77	0,15	-3,31	3,32	0,32

Supplementary table 5 Main effect of time and interactions for the Tinnitus Functional Index (TFI) based on mixed model regression analyses. Data are provided with both MTI and BTR-NF respectively as reference groups. Since multiple imputations were used, the model include estimates (EST.), standard error (SE) and p values.

Supplementary table 6 Interactions for insomnia severity

	MTI as reference group						BTR-NF as reference group					
	ITT			PP			ITT			PP		
	**EST.**	**SE**	***P***	**EST.**	**SE**	***P***	**EST.**	**SE**	***P***	**EST.**	**SE**	***P***
**ISI**												
**Time**												
T3	-1,93	0,72	**< 0.01**	-1,90	0,72	**< 0.01**	-1,20	0,79	0,13	-1,32	0,87	0,14
T4	-1,47	0,77	0,06	-1,14	0,72	0,12	-1,45	0,82	0,08	-1,59	0,83	**0,06**
**Time x Group**												
MTI vs. ADR-NF												
T3	-0,88	1,03	0,40	-0,97	1,04	0,35						
T4	-0,40	1,09	0,72	-0,89	1,05	0,40						
MTI vs. BTR-NF												
T3	0,73	1,07	0,50	0,58	1,13	0,61						
T4	0,01	1,14	0,99	-0,45	1,09	0,68						
BTR-NF vs. ADR-NF												
T3							-0,73	1,07	0,50	-1,55	1,15	0,18
T4							-0,01	1,14	0,99	-0,44	1,12	0,70

Supplementary table 6 Interactions for Insomnia Severity Index (ISI) based on mixed model regression analyses. The ITT models included all participants, who completed the pre-intervention T1 questionnaire. The PP models included only participants with sufficient quality EEG data, and who adhered to the treatment protocol. Data are provided with both MTI and BTR-NF respectively as reference groups. Since multiple imputations were used, the model include estimates (EST.), standard error (SE) and p values.

Supplementary table 7 Interactions for depression

	MTI as reference group						BTR-NF as reference group					
	ITT			PP			ITT			PP		
	**EST.**	**SE**	***P***	**EST.**	**SE**	***P***	**EST.**	**SE**	***P***	**EST.**	**SE**	***P***
**PHQ**												
**Time**												
T3	-1,44	0,52	**< 0.01**	-1,39	0,51	**< 0.01**	-1,18	0,56	**< 0.05**	-0,95	0,60	0,12
T4	-1,33	0,57	**< 0.05**	-1,35	0,54	**< 0.05**	-1,29	0,62	**< 0.05**	-0,93	0,65	0,15
MTI vs. ADR-NF												
T3	-0,59	0,74	0,42	-0,68	0,75	0,36						
T4	0,24	0,81	0,77	0,50	0,77	0,52						
MTI vs. BTR-NF												
T3	0,27	0,76	0,72	0,44	0,79	0,58						
T4	0,04	0,84	0,96	0,42	0,84	0,62						
BTR-NF vs. ADR-NF												
T3							0,70	0,86	0,42	-1,12	0,81	0,17
T4							0,00	0,88	0,99	0,09	0,85	0,92

Supplementary table 7 Interactions for depression (PHQ-9) based on mixed model regression analyses. Data are provided with both MTI and BTR-NF respectively as reference groups. Since multiple imputations were used, the model include estimates (EST.), standard error (SE) and p values.

Supplementary table 8 Interactions for Treatment Credibility and Expectations

	MTI as reference group						BTR-NF as reference group					
	ITT			PP			ITT			PP		
	**EST.**	**SE**	***P***	**EST.**	**SE**	***P***	**EST.**	**SE**	***P***	**EST.**	**SE**	***P***
**CEQ**												
**Time**												
T3	-3,56	1,30	**< 0.01**	-3,60	1,31	**< 0.01**	-2,82	1,39	**< 0.05**	-2,80	1,38	**< 0.05**
MTI vs. ADR-NF												
T3	-0,47	1,83	0,80	-0,79	1,85	0,67						
MTI vs. BTR-NF												
T3	0,74	1,90	0,70	0,81	1,90	0,67						
BTR-NF vs. ADR-NF												
T3							-1,20	1,90	0,53	-1,60	1,90	0,40

Supplementary table 8 Interactions for treatment credibility and expectations (CEQ) based on mixed model regression analyses. Data are provided with both MTI and BTR-NF respectively as reference groups. Since multiple imputations were used, the model include estimates (EST.), standard error (SE) and p values.

Supplementary figure 1 Mental strategies

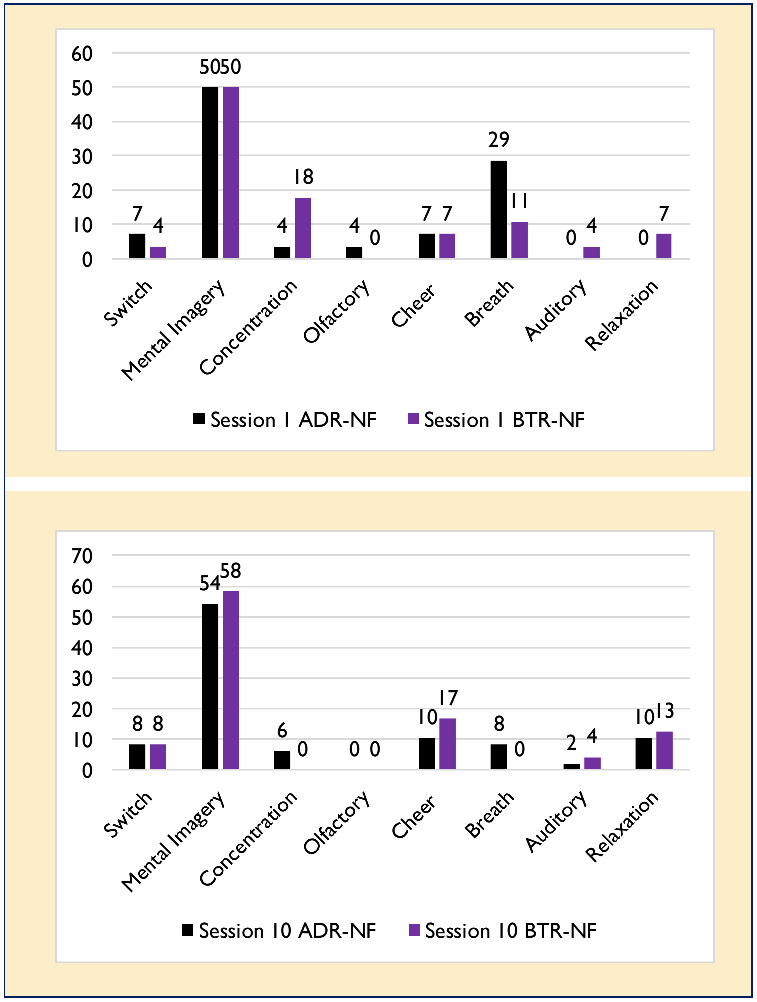

Supplementary figure 1 shows reported strategies (% within session) during neurofeedback training for session one (top panel) and ten (lower panel). Mental imagery was by far the most commonly used strategy in both groups. Classification of strategies [illegible]
